# Predictive value of drain pancreatic amylase concentration for postoperative pancreatic fistula on postoperative day 1 after pancreatic resection: An updated meta-analysis

**DOI:** 10.1097/MD.0000000000012487

**Published:** 2018-09-21

**Authors:** Yao Liu, Yang Li, Ling Wang, Ci-Jun Peng

**Affiliations:** a Department of Hepato-biliary-pancreatic Surgery; b Department of Gastroenterology, Affiliated Hospital of Zunyi Medical College, Zunyi, China.

**Keywords:** drain pancreatic amylase concentration on postoperative Day 1, meta-analysis, pancreatic resection, postoperative pancreatic fistula, predictive value

## Abstract

**Background:**

Postoperative pancreatic fistula (POPF) is a potentially fatal complication following pancreaticoduodenectomy. Early prediction and exclusion of POPF may be highly advantageous to enhance patient outcomes, and accelerate recovery. In this meta-analysis, we sought to assess the prediction of drain pancreatic amylase concentration on postoperative day 1 (DPA1) for POPF.

**Methods:**

By searching online databases up to April 2018, all researches mentioned DPA1 for detecting POPF were analyzed. STATA 12.0 was used to analyze pooled predictive parameters.

**Results:**

Seventeen studies were finally analyzed including 4676 patients in total. The pooled sensitivity and specificity of DPA1 were respectively 0.85 (95% CI: 0.71, 0.93), 0.80 (95% CI: 0.74, 0.85) to predict overall POPF, and 0.70 (95% CI: 0.53, 0.82), 0.88 (95% CI: 0.86, 0.90) to predict CR-POPF. If pretest probability was 50%, corresponding post-test (+) were respectively 81%, 86% for overall POPF and CR-POPF when DPA1 was above cutoffs, while the post-test (−) were respectively 16%, 26% when DPA1 was under cutoffs. In subgroup analysis, sensitivities of cutoff >5000 group, 1000< cutoff <5000 group, and cutoff <1000 group were respectively 0.65 (0.43–0.82), 0.82 (0.71–0.89), 0.87 (0.78–0.92); and specificities were respectively 0.88 (0.83–0.92), 0.83 (0.77–0.88), 0.71 (0.62–0.79). Positive LR was 5.5 (3.4–8.8), 4.8 (3.4–6.7), and 3.0 (2.3–4.0) respectively. Negative LR was 0.40 (0.22–0.72), 0.22 (0.13–0.37), and 0.19 (0.11–0.32) respectively.

**Conclusion:**

DPA1, which has good sensitivity and specificity, is useful for predicting overall POPF and CR-POPF, according to the present studies. Meanwhile, it should be cautious to apply because there is a wide range in cutoffs between different studies.

## Abstract 2

Supplemental Digital Content is available in the text

## 1 Introduction

Although perioperative management of patients undergoing pancreatoduodenectomy (PD) has been improved, morbidity still ranges from 20% to 50%.^[1–4]^

Postoperative pancreatic fistula (POPF), which develops in a range from 16% to 28% of patients undergoing PD, remains the major fatal complication.^[5–7]^ POPF was classified to grades A, B, and C by the International Study Group on Pancreatic Fistula (ISGPF).^[8]^ Grade A POPF is biochemical fistula, which does not have any adverse consequences. Grade B and C fistulas are generally designated as clinically relevant POPF (CR-POPF), which usually demand percutaneous drainage and hardly, laparotomy.^[8]^

So far, it has been proved that early drain removal (postoperative day (POD) <4) decreases incidence of complications, compared with late drain removal (POD >5).^[9]^ Usually, drains will be removed according to surgeon's discretion after excluding the risk of POPF. The prognosis of POPF can be advantageous to manage drains removal, enhance recovery pathway, and promote hospital discharge.^[9,10]^

Recently, many studies show high interests in drain pancreatic amylase concentration on POD 1 (DPA1) for the prediction of POPF. Although DPA1 has been implied with superb specificity and sensitivity for overall POPF and CR-POPF, there is still controversy in inconsistent opinions. The present meta-analysis especially aims to assess the value of DPA1 to predict POPF after PD.

## 2 Methods

The PRISMA statement and appropriate methods for meta-analysis were followed.^[11,12]^ The ethical statement is not necessary for this meta-analysis.

### 2.1 Study selection

MEDLINE (PubMed), Cochrane Database, Embase, and other databases were retrieved to find out corresponding papers published until April 2018. The following items were searched: “pancreatectomy,” “pancreaticoduodenectomy,” “whipple ,” “pancreatic resection,” “pancreatic fistula,” “drain amylase,” “intraperitoneal drainage amylase,” “early drain removal,” “sensitivity and specificity.” Two researchers independently reviewed the articles. When disagreements appeared, a final consensus was reached after arguing with each other.

### 2.2 Inclusion and exclusion criteria

Studies were selected by inclusion criteria as follows: prediction of DPA1 for POPF after PD; POPF recorded and defined as grade A, B, and C according to ISGPF; articles published in English language in peer-reviewed journals. Editorials, case reports, expert opinions, letters, abstracts, and studies without sufficient data to assess predictive value of DPA1 were excluded.

### 2.3 Quality assessment

The QUADAS criteria was accorded to evaluate qualities of involved studies.^[13]^

### 2.4 Data collection and statistical analysis

Data including sensitivity, specificity, and cutoff values of DPA1 for the prediction of POPF or CR-POPF were documented. STATA 12.0 was used for statistical analysis. The following figures were calculated: sensitivity, specificity, positive likelihood ratio (LR), negative LR (with corresponding 95% confidence interval), pretest probabilities, corresponding post-test probabilities, Cochran Q test, inconsistency index (I^2^), and area under the receiver operating characteristic curve (AUROC). Deeks funnel plot asymmetry test was performed to assess publication bias.

Two investigators (Y Liu and Y Li) independently extracted the data, and disagreements were settled by discussion with each other.

## 3 Results

### 3.1 Description of studies

**Figure 1:**
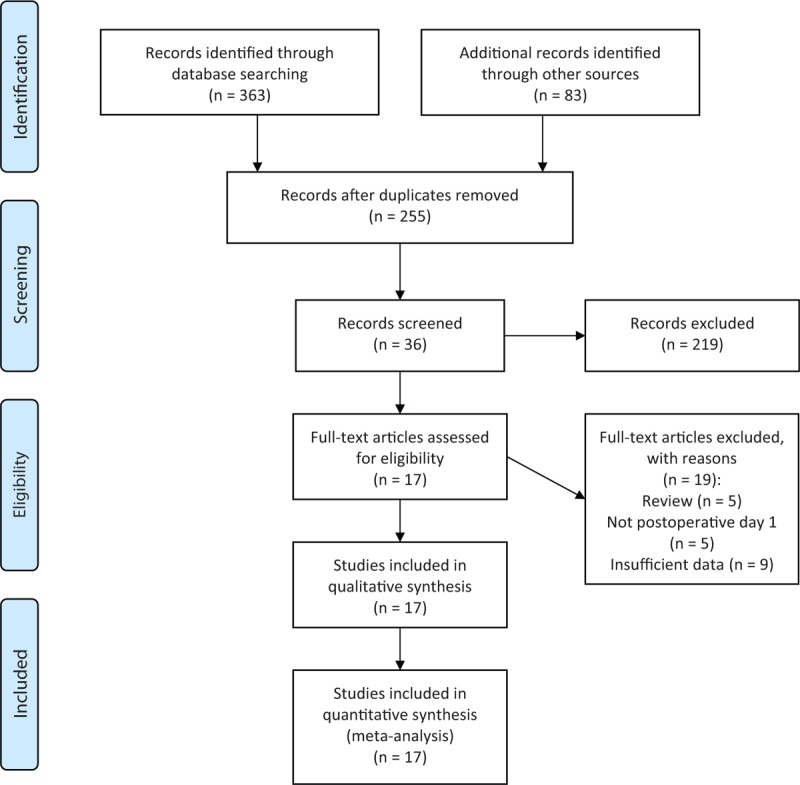
Flowchart showing literature search and selection.

Finally, 17 articles with a total of 4676 patients in 8 countries were involved.^[5,14–29]^ The procedure of selection is illustrated in Fig. 1. The information of the involved 17 studies is displayed in Table 1. There are 11 prospective and 6 retrospective studies. Diagnostic index of studies assessing prediction of DPA1 for overall POPF and CR-POPF is integrated in Table 2. Twelve studies supplied cutoffs for overall POPF, extending from 100 to 5000 U/L, while 6 for CR-POPF ranging from 1000 to 5000 U/L. And there were no significantly threshold effects of DPA1 for overall POPF and CR-POPF.

**Table 1:**
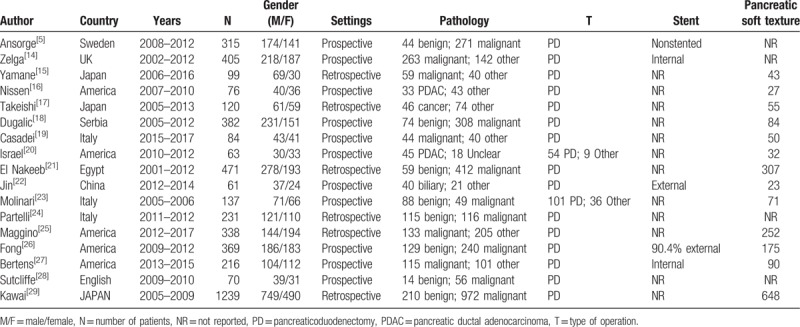
Characteristics of the selected studies.

**Table 2:**
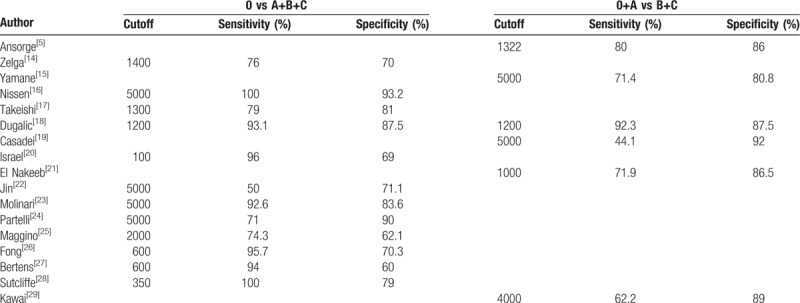
Diagnostic data evaluating DPA1 for overall POPF (0 vs A+B+C) and CR-POPF (0+A vs B+C).

### 3.2 Overall diagnostic indices

**Figure 2:**
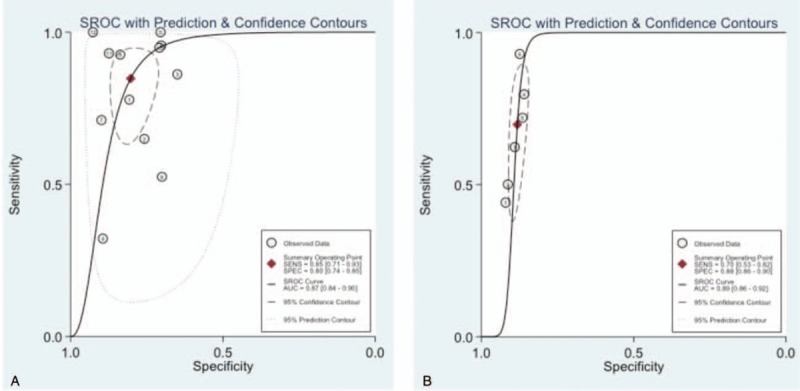
ROC curve analysis of DPA1 for the prediction of POPF (A) overall POPF: AUROC = 0.87, 95% CI (0.84, 0.90). B, CR-POPF: AUROC = 0.89, 95% CI (0.86, 0.92). AUROC = area under receiver operating characteristic, ROC =  receiver operating characteristic, CR = clinically relevant, DPA1 = drain pancreatic amylase concentration on postoperative day 1, POPF = postoperative pancreatic fistula.

Sensitivity and specificity of DPA1 for overall POPF and CR-POPF are displayed in Table 3. For predicting overall POPF, sensitivity and specificity of DPA1 were respectively 0.85 (0.71–0.93) and 0.80 (0.74–0.85), while positive and negative LR were respectively 4.30 (3.24–5.70) and 0.19 (0.10–0.37). AUROC, which was 0.87 (0.84–0.90), is illustrated in Fig. 2A. Cochran Q test showed there was significant heterogeneity of DAP1 (I^2^ = 97.28%, *P* < .001), which implied nonthreshold effects.

**Table 3:**
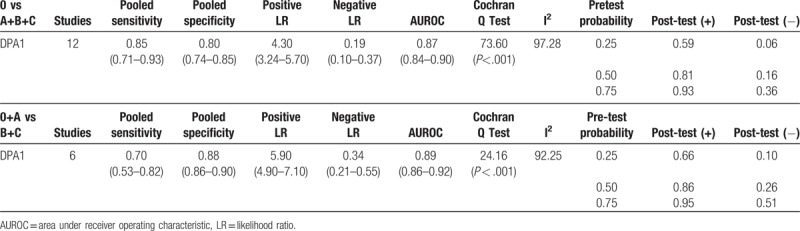
Meta-analysis of predictive data for overall POPF and CR-POPF.

For predicting CR-POPF, sensitivity and specificity of DPA1 were respectively 0.70 (0.53–0.82) and 0.88 (0.86–0.90). Positive LR, negative LR, and AUROC were respectively 5.90 (4.90–7.10), 0.34 (0.21–0.55), and 0.89 (0.86–0.92) as in Fig. 2B.

### 3.3 Fagan plot analysis

**Figure 3:**
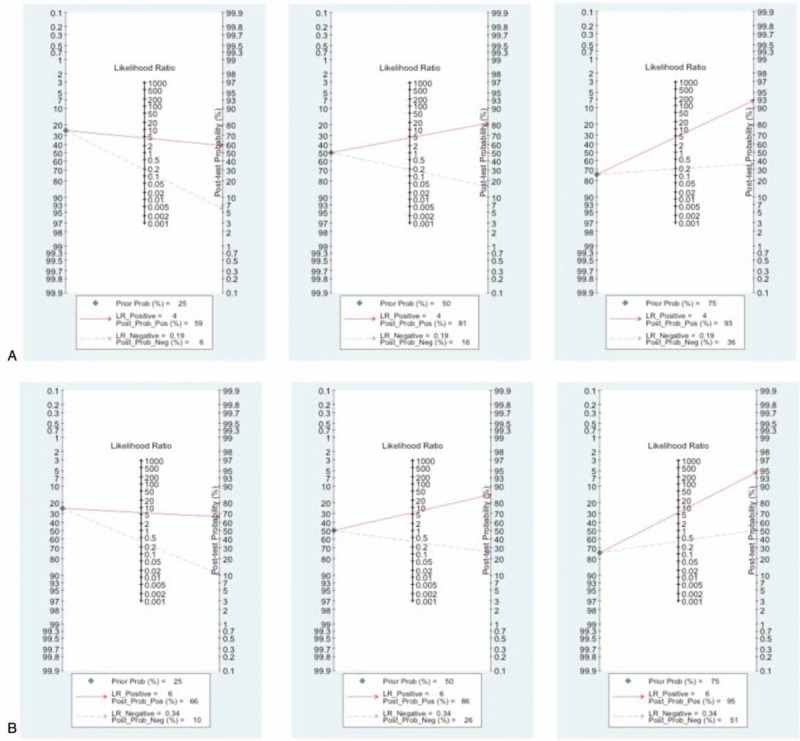
Fagan plot for the assessment of clinical application (A) DPA1 for overall POPF. B, DPA1 for CR-POPF. CR =  clinically relevant, DPA1 = drain pancreatic amylase concentration on postoperative day 1, POPF = postoperative pancreatic fistula.

In Fig. 3, the Fagan plot in DPA1 for overall POPF^[14,16–18,20,22–28]^ implied that when the pretest probabilities were respectively 25%, 50%, 75% the positive post-test probabilities (post-test (+)) were 0.59, 0.81, 0.93 and the negative post-test probabilities (post-test (−)) were 0.06, 0.16, 0.36. For CR-POPF,^[5,15,18,19,21,29]^ when the pretest probabilities were respectively 25%, 50%, 75% the post-tests (+) were 0.66, 0.86, 0.95, and the post-tests (−) were 0.10, 0.26, 0.51.

### 3.4 Meta-regression and subgroup analysis

For the wide range of cutoffs between studies when predicting overall POPF in DPA1, meta-regression analysis was applied to find the heterogeneity sources. The “country,” “study setting,” “stent,” “type of operation,” and “pancreatic soft texture” were involved. According to the results, the main sources of heterogeneity were country, type of operation, and pancreatic soft texture.

Huge differences of various cutoffs existed in the involved studies, and then subgroup analysis was performed. Results are demonstrated in supplementary Table 1. The summary sensitivities of cutoff ≤1000 group, 1000< cutoff <5000 group, and cutoff ≥ 5000 group were respectively 0.87 (0.78–0.92), 0.82 (0.71–0.89), and 0.65 (0.43–0.82); the summary specificities were respectively 0.71 (0.62–0.79), 0.83 (0.77–0.88), and 0.88 (0.83–0.92). Positive LR were respectively 3.0 (2.3–4.0), 4.8 (3.4–6.7), and 5.5 (3.4–8.8). Negative LR were respectively 0.19 (0.11–0.32), 0.22 (0.13–0.37), and 0.40 (0.22–0.72). AUROC were respectively 0.86 (0.83–0.89), 0.89 (0.86–0.91), and 0.89 (0.86–0.91). The results of Fagan plot analysis show that, in cutoff ≥5000 group, when the pretest probabilities were respectively 25%, 50%, 75%, post-test (+) were 0.65, 0.85, 0.94, and post-test (−) were 0.12, 0.28, 0.54; in 1000< cutoff <5000 group, post-test (+) were 0.61, 0.83, 0.93, and post-test (−) were 0.07, 0.18, 0.40; and in cutoff ≤1000 group, post-test (+) were 0.50, 0.75, 0.90, and post-test (−) were 0.06, 0.16, 0.36.

**Figure 4:**
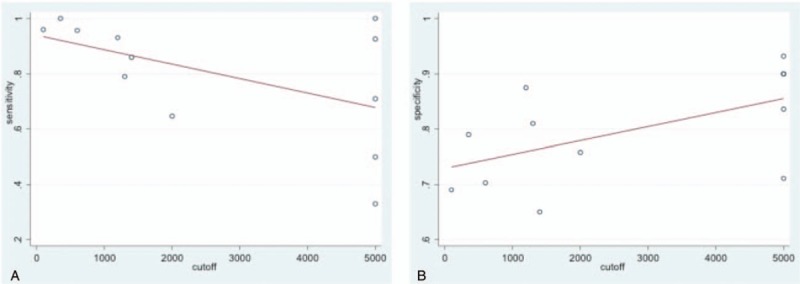
Meta regression of DPA1 for overall POPF (A) sensitivity. B, Specificity. DPA1 = drain pancreatic amylase concentration on postoperative day 1, POPF = postoperative pancreatic fistula.

Meta regression was applied to estimate overall sensitivity and specificity, using the various cutoffs among studies as an independent predictor in Fig. 4.

### 3.5 Publication bias

**Figure 5:**
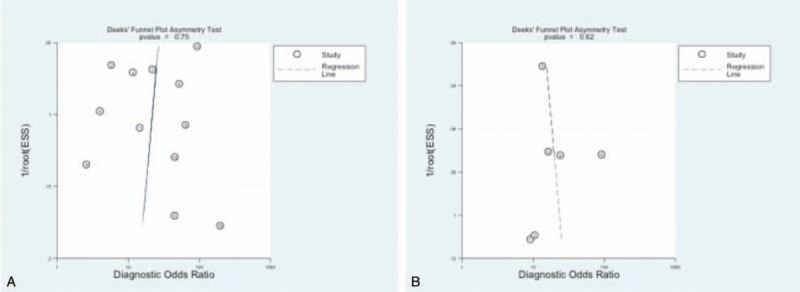
Assessment of publication bias (A) overall POPF. B, CR-POPF. CR =  clinically relevant, POPF = postoperative pancreatic fistula.

Deeks funnel plot asymmetry test indicates there is no publication bias among the studies in Fig. 5.

## 4 Discussion

POPF is still a potentially fatal complication, which may increase financial utilization after pancreatic resection. There already arise controversies about intraperitoneal drains following PD. In a recent study,^[29]^ it indicated that early drains removal (POD 4) had significant benefits on decreasing incidence of POPF. Furthermore, in a prospective study with 84 patients who were performed PD, it revealed that it is safe to pull out drains on POD 3 following PD with a lower incidence of POPF in patients with DPA1 ≤5000 U/L.^[19]^

Early prediction of POPF can significantly benefit the patient following PD; however, few studies have assessed the predictive accuracy of DPA for developing of POPF.

Several markers, such as DPA, CRP, WBC, have been proposed as predictors for POPF.^[5–7,28,30,31]^ It was implied by Molinari et al^[23]^ that DPA1 > 5000 U/L had a respectively sensitivity and specificity of 93% and 84% for the prediction of POPF following PD. Besides, Ansorge et al^[5]^ recommended serum CRP association with DPA to predict CR-POPF. With the comprehensive consideration, this meta-analysis aimed to assess the accuracy of DPA1 for the prediction of POPF. Up to now, there are few studies to assess the pooled performance of DAP1 for overall and clinically relevant POPF.

In this meta-analysis, DPA1 displayed an outstanding capability in identifying POPF with a high positive LR, which could be acted as a rule-in means for the diagnosis of POPF. Meanwhile, it also showed an acceptable sensitivity and specificity. When the pretest probability was set at 50%, DPA1 indicated an accurate diagnosis of overall POPF in 81% patients and misdiagnosis only in 16% patients by Fagan plot analysis, besides, it also showed accuracy for CR-POPF in 86% positive patients and misdiagnosis in 26% patients. With comprehensive consideration of the pooled results, DPA1 is an appropriate marker for the prediction of POPF. Certainly, more randomized controlled trials should be implemented to provide evidence.

In the present study, it supplies beneficial information to help researchers and clinicians to predict POPF by DPA1. However, there are several limitations in this meta-analysis. First, few studies are assessed as high quality to offer unbiased data. Second, studies involved in this meta-analysis had a vast range in cut-off values. Besides, few studies provided specific amylase range or cutoff at each grade of POPF. Therefore, it is necessary to further explore the values of DPA1 and other markers for predicting the grade of POPF in randomized studies.

It is concluded that DPA1 is a valuable marker to predict POPF, and more randomized controlled trials should be implemented to provide unbiased evidences.

## Acknowledgment

The authors thank Prof Yuan-Zhong Zhou from Public Health Institute, Zunyi Medical College for statistical consultation.

## Author contributions

Y Liu proposed the study. Y Liu and Y Li performed research, collected the data, and wrote the first draft. Y Li and LW analyzed the data. C-JP revised the manuscript. All authors contributed to the design and to further drafts.

**Data curation:** Yao Liu, Yang Li, Ling Wang.

**Software:** Ling Wang.

**Writing – original draft:** Yao Liu, Yang Li.

**Writing – review & editing:** Ci-Jun Peng.

## Supplementary Material

Supplemental Digital Content
