# Supplementary material for: Predictive value of drain pancreatic amylase concentration for postoperative pancreatic fistula on postoperative day 1 after pancreatic resection: An updated meta-analysis
Source: Medicine (Baltimore). 2018 Sep 21;97(38):e12487. doi: 10.1097/MD.0000000000012487 (PMC6160246; doi:10.1097/MD.0000000000012487)
Supplement: Supplemental Digital Content [file medi-97-e12487-s001.doc]

Supplementary Table 1 Meta-analysis of predictive data for POPF

| Cutoff | Studies | Pooled | Pooled | Positive | Negative | AUROC | Cochran’s | I^2^ | Pre-test | Post-test (+) | Post-test (-) |
| --- | --- | --- | --- | --- | --- | --- | --- | --- | --- | --- | --- |
|  |  | Sensitivity | Specificity | LR | LR |  | Q Test |  | probability |  |  |
| ≥5000 | 7 | 0.65 | 0.88 | 5.50 | 0.40 | 0.89 | 17.108 | 88.0 | 0.25 | 0.65 | 0.12 |
|  |  | (0.43-0.82) | (0.83-0.92) | (3.40-8.80) | (0.22-0.72) | (0.86-0.91) |  |  |  |  |  |
|  |  |  |  |  |  |  |  |  | 0.50 | 0.85 | 0.28 |
|  |  |  |  |  |  |  |  |  | 0.75 | 0.94 | 0.54 |
| 1000-5000 | 7 | 0.82 | 0.83 | 4.80 | 0.22 | 0.89 | 73.226 | 97.0 | 0.25 | 0.61 | 0.07 |
|  |  | (0.71-0.89) | (0.77-0.88) | (3.40-6.70) | (0.13-0.37) | (0.86-0.91) |  |  |  |  |  |
|  |  |  |  |  |  |  |  |  | 0.50 | 0.83 | 0.18 |
|  |  |  |  |  |  |  |  |  | 0.75 | 0.93 | 0.40 |
| ≤1000 | 4 | 0.87 | 0.71 | 3.0 | 0.19 | 0.86 | 20.545 | 90.0 | 0.25 | 0.50 | 0.06 |
|  |  | (0.78-0.92) | (0.62-0.79) | (2.30-4.00) | (0.11-0.32) | (0.83-0.89) |  |  |  |  |  |
|  |  |  |  |  |  |  |  |  | 0.50 | 0.75 | 0.16 |
|  |  |  |  |  |  |  |  |  | 0.75 | 0.90 | 0.36 |

LR, likelihood ratio; AUROC, area under receiver operating characteristic.

A, assessment of publication bias of involved studies

B, assessment of clinical application

Supplementary Figure 1. The QUADAS method to assess quality of involved studies.
